# Data on the physical and mechanical properties of soilcrete materials modified with metakaolin

**DOI:** 10.1016/j.dib.2017.06.014

**Published:** 2017-06-10

**Authors:** Panagiotis G. Asteris, Konstantinos G. Kolovos

**Affiliations:** a Computational Mechanics Laboratory, Department of Civil Engineering, School of Pedagogical & Technological Education, Athens, Greece; b Department of Military Sciences, Division of Physical Sciences and Applications, Hellenic Army Academy, Vari, Greece

**Keywords:** Soilcrete materials, Metakaolin, Concrete technology, Physical and mechanical properties, Compressive strength, Modulus of elasticity, Ultrasonic pulse velocity, Artificial neural networks

## Abstract

During the last decades eco-friendly, low-cost, sustainable construction materials for utilization in civil engineering projects have attracted much attention. To this end, soilcretes are non-conventional construction materials produced by mixing natural soil such as natural clay or limestone sand with a hydraulic binder and are recently under detailed and in-depth investigation by many researchers. In this paper the results of the physical and mechanical characteristics of a large set of cylindrical specimens under uniaxial compression, are presented. Specifically, two types of soils such as sand and clay with metakaolin as a mineral additive have been used. This database can be extremely valuable for better understanding of the behavior of soilcrete materials. Furthermore, the results presented herein expected to be of great interest for researchers who deal with the prediction of mechanical properties of materials using soft computing techniques such as artificial intelligence (AI) techniques.

## Specifications Table

**Table:** 

Subject area	*Materials Science and Engineering*
More specific subject area	*Construction and Building materials*
Type of data	*Tables, figure*
How the data were acquired	*Macroscopical and experimental tests*
Data format	*Raw, analyzed*
Experimental factors	*Pretreatment of samples included drying of natural clay ground soil at 105 °C for 5 days and crushed quarry sand at 105 °C for 24 h in an electrical laboratory oven and then sieved in order to pass a 2 and 4.75 mm sieve correspondingly. Mixing of binder batches was conducted in a laboratory swing mill for 1 h without further grinding of solid constituents until homogeneity of the blends was reached.*
Experimental features	*Testing the shrinkage, bulk density after 1 day of curing and compressive strength, modulus of elasticity and strain at maximum stress after 28 days of curing of 2 type soilcrete materials samples that contained different contents of natural clay ground soil or crushed quarry sand of a limestone origin and different content of a variable-in-synthesis ordinary Portland cement-metakaolin binder at different water/binder ratio values in laboratory situation*
Data source location	*Laboratory of Concrete & Aseismic Constructions, Department of Civil Engineering, School of Pedagogical & Technological Education, Athens, Greece*
Data accessibility	*Data within this article*

## Value of the data

• The research data are important for researchers who deal with eco-friendly, low-cost, new, sustainable construction materials with improved mechanical properties for utilization in civil engineering projects.
• The experimental data can be extremely valuable for the development of a mix design process focused on soilcrete materials and to elucidate the relation between physical and mechanical properties through the comparison of macroscopical observations with experimentally measured mechanical characteristics.
• The experimental data presented herein expected to be of great interest for researchers who deal with the prediction of mechanical properties of materials using soft computing techniques such as artificial intelligence (AI) techniques and provide an initial dataset point to generate and validate numerical and/or analytical models.

## 1 Data

**Fig. 1:**
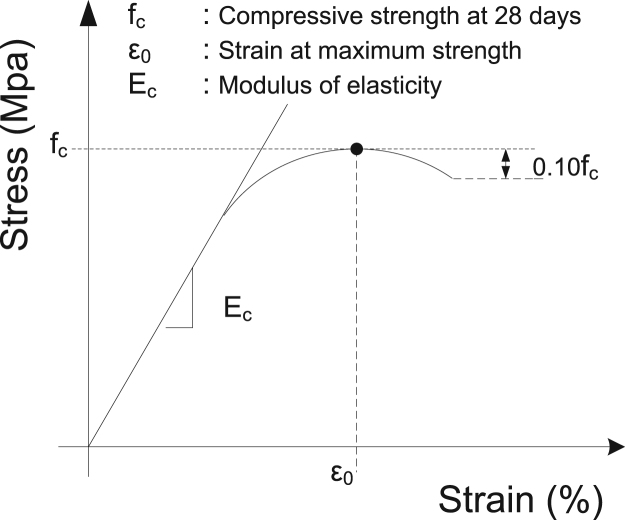
Stress–strain curves.

Data on the synthesis and physicomechanical characteristics of 2 types of environment-friendly soilcrete materials are presented. Soilcrete samples contained different contents of natural clay ground soil or crushed quarry sand of a limestone origin as replacement of the aggregate phase. Metakaolin has been added at variable contents as a mineral additive to the ordinary Portland cement-based binder mix, at different water/binder ratio values (*W/B*). Specifically, the Research Database presents measured physical and mechanical properties such as the 28 days compressive strength (*f*_*c*_), the modulus of elasticity (*E*_*c*_) and the strain at maximum strength (*ε*_*0*_) (Fig. 1) of a large set of cylindrical specimens with a height-to-diameter (*h/d*) ratio equal to 2 (*h/d*=2) which have been tested under uniaxial compression [1], [2], [3].

## 2 Experimental design

Three categories of binders (B), variable in synthesis, were investigated; the 1st that consisted of 100% w/w CEM I 42.5  N Portland cement (PC) used as reference, the 2nd produced by mixing 90% w/w PC and 10% w/w metakaolin (MK) in the dry mix and the 3rd by mixing 80% w/w PC and 20% w/w MK (in the dry mix) as partial replacement. Homogeneity of all 3 categories of blends was reached after mixing MK and PC without further grinding in a laboratory swing mill for 1 h [1], [2], [3].

Batches of samples that correspond to 2 specific types of soil materials were investigated; natural clay ground soil (CGS) and crushed quarry limestone sand (CQLS) were used as replacement of the aggregate phase. First type soilcrete material, entitled as clay-based soilcrete, produced by mixing 50% and 70% w/w CGS (in the dry mix) with 50% and 30% w/w binder at 2 different water/binder (*W/B*) ratio values of 0.48 and 1.2. High workability and optimal flow properties for samples with *W/B* equal to 0.48 was achieved by the addition of 2.0% w/w (of the cementitious materials) superplasticizer (SP), as presented in Table 1. Second type soilcrete material, entitled as sand-based soilcrete, produced by mixing 50% and 70% w/w CQLS (in the dry mix) with 50% and 30% w/w binder at 3 different *W/B* ratio values of 0.4, 0.7 and 1.0. As Similarly to the first type soilcrete material samples, high workability and optimal flow properties for samples with *W/B* equal to 0.4 were achieved by the addition of 2.0% w/w SP (of the cementitious materials), as listed in Table 2. Refs. [1], [2], [3] provide a detailed description of the experimental set-up.

**Table 1:** Synthesis and experimental design of clay-based soilcrete material (first sheet of the MS-Excel file under the title “Database of SoilCrete Materials” in the Supplementary document).

**Sample**	**CEM Ι 42.5 N**	**Μetakaolin**	**Clay ground soil**	***W/B***	**Superplasticizer**
	**(PC)**	**(MK)**	**(CGS)**		**(SP)**
	**(% w/w in the dry mix)**	**(% w/w in the dry mix)**	**(% w/w in the dry mix)**		**(% w/w of the cementitious materials)**
PC-50L	50	–	50	0.48	2.0
PC-30L	30	–	70	0.48	2.0
PMC10-50L	45	5	50	0.48	2.0
PMC10-30L	27	3	70	0.48	2.0
PMC20-50L	40	10	50	0.48	2.0
PMC20-30L	24	6	70	0.48	2.0
PC-50H	50	–	50	1.2	–
PC-30H	30	–	70	1.2	–
PMC10-50H	45	5	50	1.2	–
PMC10-30H	27	3	70	1.2	–
PMC20-50H	40	10	50	1.2	–
PMC20-30H	24	6	70	1.2	–

**Table 2:** Synthesis and experimental design of sand-based soilcrete material (second sheet of the MS-Excel file under the title “Database of SoilCrete Materials” in the Supplementary document).

**Sample**	**CEM Ι 42.5 N**	**Μetakaolin**	**Crushed Quarry Limestone Sand**	***W/B***	**Superplasticizer**
	**(PC)**	**(MK)**	**(CQLS)**		**(SP)**
	**(% w/w in the dry mix)**	**(% w/w in the dry mix)**	**(% w/w in the dry mix)**		**(% w/w of the cementitious materials)**
PCSA–50L	50	–	50	0.4	2.0
PCSA–30L	30	–	70	0.4	2.0
PMCSA10–50L	45	5	50	0.4	2.0
PMCSA10–30L	27	3	70	0.4	2.0
PMCSA20–50L	40	10	50	0.4	2.0
PMCSA20–30L	24	6	70	0.4	2.0
PCSA–50M	50	–	50	0.7	–
PCSA–30M	30	–	70	0.7	–
PMCSA10–50M	45	5	50	0.7	–
PMCSA10–30M	27	3	70	0.7	–
PMCSA20–50M	40	10	50	0.7	–
PMCSA20–30M	24	6	70	0.7	–
PCSA–50H	50	–	50	1.0	–
PCSA–30H	30	–	70	1.0	–
PMCSA10–50H	45	5	50	1.0	–
PMCSA10–30H	27	3	70	1.0	–
PMCSA20–50H	40	10	50	1.0	–
PMCSA20–30H	24	6	70	1.0	–

## 3 Materials

Raw materials used for the preparation of samples were:

(i) Type CEM I 42.5 N ordinary Portland cement (PC) of industrial origin to avoid the additional influence of the presence of mineral admixtures found in other CEM types, to minimize water demand and to comply with requirements concerning initial strength development, according to EN 197-1 [1], [2], [3].
(ii) High purity commercial metakaolin (MK) supplied by Imerys Minerals, containing 95% w/w metakaolinite (Al_2_O_3_·2SiO_2_) [1], [2], [3].
(iii) Natural clay ground soil (CGS) since it corresponds to a typical worst case scenario of a low plasticity stiff soil [1].
(iv) Crushed quarry sand of a limestone origin (CQLS) was selected since it corresponds to a widespread typical natural rock that can be found in a wide range of soil strata (clean sands) [1], [3].
(v) Type E and F according to ASTM C494/C494M-08a common superplasticizer (SP) manufactured by Domylco Ltd. (CHEM SLP P) at appropriate percentages in order to retain high workability and optimal flow properties of the produced soilcrete.
(vi) Tap water (W) of moderate hardness (14 °F).

## 4 Methods

Particle size distribution for CGS (before sieving at 2 mm) and CQLS (before sieving at 4.75 mm) resulted by conducting sieve analysis according to ASTM D6913-04, ASTM E11 and C136 standards, respectively [1], [2], [3]. CGS and CQLS were dried at 105 °C for 5 days and at 105 °C for 24 h respectively, in an electrical laboratory oven and then sieved in order to pass a 2.00 and 4.75 mm sieve respectively [1], [2], [3]. Mixing of binder batches was performed in a laboratory swing mill for 1 h without further grinding of solid constituents until homogeneity of the blends was reached [1], [2], [3]. Soilcrete samples were prepared by mixing the binder-CGS and binder-CQLS mixtures with tap water at 20 °C in an 80 L capacity laboratory mixer used for concrete production [1], [2], [3].

All soilcrete specimens were cast in concrete cylinders (100 mm in diameter, 200 mm in high), vibrated for 20 s on a vibration table and then covered to minimize water evaporation [1], [2], [3]. The molds were stripped after 24 h, and the specimens were immersed in lime-saturated water at 20 °C, until testing [1], [2], [3]. Compressive strength measurements were conducted at 28 days. For each sample, four to eight soilcrete specimens were tested and the mean values of the measured physical and mechanical properties such as the 28 days compressive strength, the modulus of elasticity, the strain at maximum strength and the ultrasonic velocity are presented at Table 3, Table 4.

**Table 3:** Physical and mechanical measured characteristics of clay-based soilcrete material samples (third sheet of the MS-Excel file under the title “Database of SoilCrete Materials” in the Supplementary document).

**Soil type**	**Specimen no.**	**W/B ratio**	**MK (% w/w in the dry mix)**	**B (% w/w in the dry mix)**	**SP (% w/w of the cementitious materials)**	**Shrinkage (%)**	**Density (kg/m**^**3**^**)**	**Ultrasonic velocity (m/s)**	**Compressive strength (MPa)**	**Modulus of elasticity (GPa)**	**Strain at maximum stress**
**CGS**	1	0.48	0	50	2.0	7.5	2089.2	2059.03	39.31	5.979	0.00695222
	2					3.5	2142.5	2002.68	37.08	5.579	0.00751251
	3					5.5	2102.9	2025.74	37.76	5.379	0.00827908
	4					4.0	2074.0	1986.76	32.06	5.082	0.00612117
	5					5.0	2161.6	1998.67	32.84	5.744	0.00672800
	1	0.48	0	30	2.0	7.5	2082.3	1974.99	44.61	6.458	0.00567568
	2					4.0	2046.2	1921.89	33.63	6.054	0.00791667
	3					3.5	2139.9	1940.52	41.10	6.250	0.00765230
	4					9.0	2041.0	2035.28	51.07	6.641	0.00807700
	5					4.5	2143.6	1954.42	47.17	6.391	0.00743455
	1	0.48	5.0	50	2.0	7.5	2078.2	3585.39	54.65	7.067	0.00766000
	2					8.0	2041.0	3747.67	53.25	7.066	0.00447970
	3					5.0	2078.4	3668.07	57.55	7.762	0.00785000
	4					6.0	2024.7	3698.45	45.96	7.310	0.00660000
	1	0.48	3.0	30	2.0	5.0	1944.4	3475.65	40.51	6.120	0.00645860
	2					5.5	2004.5	3600.14	58.94	6.827	0.00884138
	3					5.0	2091.9	3553.80	46.20	6.649	0.00780800
	4					7.0	1962.9	3579.30	38.56	5.397	0.00775800
	5					4.5	2004.9	3647.41	34.90	5.833	0.00594590
	1	1.2	0	50	0	19.0	1706.4	2781.92	15.41	2.716	0.00884200
	2					16.5	1682.7	2739.20	18.59	2.737	0.00960100
	3					15.0	1668.0	2788.42	15.07	2.845	0.00724588
	4					18.0	1637.4	1654.72	15.07	3.003	0.00870000
	5					19.0	1701.7	1692.05	21.16	2.733	0.00864470
	1	1.2	0	30	0	17.5	1752.6	1679.75	24.54	3.259	0.00918227
	2					16.5	1733.1	1635.78	23.01	3.131	0.00892216
	3					16.5	1762.1	1637.57	21.61	2.701	0.00982000
	4					16.5	1719.4	1640.24	22.92	2.851	0.00910180
	5					15.5	1729.2	1648.36	22.56	3.075	0.00840237
	1	1.2	5.0	50	0	7.5	1528.7	2560.55	16.82	2.751	0.00720000
	2					11.0	1614.5	2709.29	18.01	2.980	0.00720000
	3					8.5	1683.2	2716.51	19.08	3.355	0.00732000
	4					9.0	1640.7	2688.33	22.55	3.470	0.00758900
	1	1.2	3.0	30	0	10.0	1685.8	2787.84	15.41	2.775	0.00695000
	2					10.0	1702.8	2776.38	18.59	3.379	0.00689500
	3					7.5	1662.2	2808.73	15.07	1.995	0.00757100
	4					7.5	1714.6	2740.77	15.07	1.974	0.00760000
	5					7.5	1603.0	2769.47	21.16	3.161	0.00660400

**Table 4:** Physical and mechanical measured characteristics of sand-based soilcrete material samples (fourth sheet of the MS-Excel file under the title “Database of SoilCrete Materials” in the Supplementary document).

**Soil type**	**Specimen no.**	**W/B ratio**	**MK (% w/w in the dry mix)**	**B (% w/w in the dry mix)**	**SP (% w/w of the cementitious materials)**	**Shrinkage (%)**	**Density (kg/m**^**3**^**)**	**Ultrasonic velocity (m/s)**	**Compressive strength (MPa)**	**Modulus of elasticity (GPa)**	**Strain at maximum stress**
**CQLS**	1	0.4	0	50	2.0	1.0	2111.9	4070.00	55.35	27.442	0.00290000
	2					1.0	2100.8	4016.67	62.25	24.325	0.00440000
	3					1.0	2130.2	4053.33	41.04	24.875	0.00074000
	4					0.5	2146.4	4100.00	58.00	27.754	0.00300000
	5					0.5	2125.6	4076.67	50.35	27.249	0.00110000
	6					1.5	2123.6	4040.00	46.48	26.476	0.00460000
	7					1.0	2121.9	4090.00	61.49	28.976	0.00280000
	8					1.0	2100.8	4016.67	62.25	24.325	0.00440000
	1	0.4	0	30	2.0	3.5	2161.4	4006.67	62.35	25.690	0.00340000
	2					3.0	2161.3	4080.00	66.72	25.765	0.00440000
	3					3.3	2186.2	4040.00	57.17	29.371	0.00260000
	4					3.0	2173.2	4100.00	60.79	25.679	0.00290000
	5					3.0	2176.8	4000.00	50.36	25.650	0.00320000
	6					2.5	2154.5	4070.00	64.64	27.577	0.00350000
	7					3.3	2186.2	4040.00	57.17	29.371	0.00260000
	8					2.5	2161.7	4063.33	50.66	28.145	0.00200000
	1	0.4	5.0	50	2.0	1.0	2077.8	3913.33	49.36	24.745	0.00130000
	2					0.3	2070.1	3931.67	48.30	24.160	0.00315000
	3					1.0	2074.6	3916.67	48.86	28.668	0.00210000
	4					1.0	2059.1	3980.00	49.01	26.747	0.00190000
	5					0.0	2076.4	3840.00	41.86	23.543	0.00270000
	6					0.0	2075.8	3900.00	39.87	28.434	0.00210000
	7					0.3	2070.1	3931.67	48.30	24.160	0.00315000
	8					2.5	2098.0	3810.00	59.82	26.746	0.00260000
	1	0.4	3.0	30	2.0	0.5	2128.8	4090.00	62.01	28.182	0.00280000
	2					1.0	2129.6	4053.33	59.44	28.644	0.00275000
	3					1.0	2129.6	4053.33	59.44	28.644	0.00275000
	4					1.0	2137.3	4070.00	58.03	28.360	0.00240000
	5					1.5	2143.9	4003.33	60.87	29.478	0.00290000
	6					2.5	2132.6	3966.67	46.26	28.360	0.00180000
	7					1.0	2133.4	4023.33	63.05	29.625	0.00270000
	8					1.5	2144.6	3986.67	51.67	26.791	0.00200000
	1	0.4	10.0	50	2.0	1.5	2099.3	3926.67	76.90	26.513	0.00330000
	2					1.3	2115.6	3831.67	56.03	23.159	0.00280000
	3					1.5	2114.2	3763.33	68.21	24.276	0.00360000
	4					0.5	2101.3	3810.00	72.48	25.168	0.00320000
	5					2.0	2138.3	3873.33	68.86	26.876	0.00310000
	6					1.3	2115.6	3831.67	56.03	23.159	0.00280000
	7					1.5	2117.1	3746.67	71.26	23.733	0.00340000
	8					1.0	2131.8	3756.67	71.57	27.914	0.00340000
	1	0.4	6.0	30	2.0	1.8	2102.5	3886.67	64.65	25.695	0.00330000
	2					2.5	2093.4	3820.00	72.68	26.582	0.00350000
	3					1.0	2101.3	3906.67	74.34	26.781	0.00360000
	4					1.0	2095.2	3880.00	67.92	25.120	0.00620000
	5					3.0	2099.6	3903.33	75.77	25.578	0.00390000
	6					0.5	2102.6	3863.33	70.94	26.992	0.00350000
	7					1.8	2102.5	3886.67	64.65	25.695	0.00330000
	8					2.0	2120.1	3890.00	60.81	26.798	0.00260000
	1	0.7	0	50	0	10.0	1995.4	3523.33	27.87	15.884	0.00260000
	2					11.0	1909.4	3353.33	22.53	12.785	0.00400000
	3					10.0	1912.2	3333.33	25.16	16.654	0.00190000
	4					10.0	1934.9	3381.67	26.68	14.166	0.00362500
	5					9.5	1916.8	3356.67	25.18	13.710	0.00230000
	6					13.0	1940.8	3376.67	28.75	15.392	0.00280000
	7					10.0	1934.9	3381.67	26.68	14.166	0.00362500
	1	0.7	0	30	0	8.0	1976.9	3486.67	26.72	17.402	0.00230000
	2					8.0	2036.1	3670.00	28.63	20.870	0.00280000
	3					8.0	1979.7	3536.67	23.53	21.957	0.00170000
	4					8.5	1917.1	3343.33	26.07	14.697	0.00280000
	5					8.5	1997.1	3516.67	28.83	16.694	0.00250000
	6					8.0	1950.3	3486.67	26.44	15.622	0.00240000
	7					8.5	1969.0	3436.67	28.06	18.379	0.00230000
	8					8.5	1980.1	3413.33	33.32	15.280	0.00370000
	1	0.7	5.0	50	0	3.0	1893.4	3303.33	35.60	15.233	0.00360000
	2					4.5	1890.8	3406.67	31.48	13.865	0.00300000
	3					4.5	1922.8	3303.33	31.61	14.062	0.00290000
	4					2.3	1880.7	3333.33	32.59	13.197	0.00370000
	5					2.0	1876.7	3533.33	30.51	15.296	0.00270000
	6					3.0	1878.0	3383.33	32.99	12.730	0.00360000
	7					2.3	1880.7	3333.33	32.59	13.197	0.00370000
	8					2.5	1887.6	3373.33	32.71	13.913	0.00330000
	1	0.7	3.0	30	0	5.0	1965.8	3473.33	31.53	16.269	0.00270000
	2					3.0	1910.8	3530.00	30.69	15.240	0.00265000
	3					3.5	1940.5	3516.67	31.00	14.834	0.00380000
	4					3.0	1917.1	3473.33	29.55	14.138	0.00250000
	5					2.5	1938.6	3420.00	29.43	16.757	0.00260000
	6					5.0	1939.7	3493.33	33.11	15.356	0.00300000
	7					2.5	1935.3	3500.00	30.44	15.064	0.00290000
	8					2.5	1934.3	3446.67	35.51	14.515	0.00390000
	1	0.7	10.0	50	0	3.0	1848.4	3386.67	40.78	12.960	0.00370000
	2					3.0	1842.5	3396.67	44.13	13.138	0.00410000
	3					3.0	1857.0	3386.67	38.48	12.989	0.00350000
	4					3.0	1855.0	3416.67	38.327	14.1785	0.00315000
	5					3.0	1855.0	3416.67	38.33	14.179	0.00315000
	6					3.0	1864.9	3386.67	38.28	13.626	0.00340000
	7					3.0	1838.3	3373.33	39.71	13.640	0.00350000
	8					2.5	1842.6	3426.67	41.75	14.144	0.00360000
	1	0.7	6.0	30	0	1.5	1907.9	3473.33	35.68	15.308	0.00280000
	2					1.0	1903.4	3466.67	34.94	16.008	0.00260000
	3					1.5	1923.8	3480.00	33.27	16.529	0.00240000
	4					0.5	1876.6	3423.33	35.82	16.929	0.00270000
	5					1.0	1883.5	3456.67	39.31	16.124	0.00310000
	6					3.0	1911.5	3440.00	38.16	15.525	0.00290000
	7					1.0	1917.6	3446.67	33.79	17.725	0.00340000
	8					1.5	1894.7	3400.00	35.49	20.365	0.00250000
	1	1.0	0	50	0	17.5	1852.9	2996.67	12.21	11.080	0.00340000
	2					21.5	1884.9	3076.67	15.41	12.005	0.00990000
	3					21.5	1955.5	3216.67	17.39	15.598	0.00320000
	4					24.0	1844.6	3086.67	16.39	10.943	0.00250000
	5					26.0	1775.7	3026.67	15.05	12.352	0.00130000
	1	1.0	0	30	0	6.0	2029.1	3430.00	17.21	18.732	0.00190000
	2					18.0	1934.5	3233.33	15.52	14.212	0.00210000
	3					18.0	1929.9	3173.33	16.56	14.149	0.00190000
	4					18.0	1873.2	3083.33	15.28	12.740	0.00170000
	1	1.0	5.0	50	0	13.5	1796.0	3163.33	17.32	15.575	0.00100000
	2					13.5	1815.5	3230.00	16.03	13.224	0.00170000
	3					13.5	1696.9	3053.33	18.64	9.836	0.00280000
	4					15.5	1754.8	3180.00	17.20	11.299	0.00180000
	5					11.0	1729.4	3040.00	14.37	10.686	0.01600000
	6					11.5	1640.6	2933.33	14.67	10.341	0.00190000
	7					13.0	1676.6	3010.00	14.74	11.803	0.00140000
	1	1.0	3.0	30	0	10.5	1806.2	3116.67	16.13	12.304	0.00180000
	2					11.5	1902.2	3350.00	20.84	12.832	0.00250000
	3					9.0	1768.7	3130.00	14.28	10.094	0.00200000
	4					10.5	1724.4	2993.33	14.16	10.735	0.00210000
	5					9.0	1791.8	3180.00	14.42	10.125	0.00210000
	6					9.0	1717.3	3006.67	15.60	11.474	0.00210000
	7					10.5	1789.8	3063.33	15.74	11.834	0.00190000
	1	1.0	10.0	50	0	3.5	1652.4	2906.67	19.00	10.029	0.00310000
	2					5.5	1662.1	2983.33	20.26	9.171	0.00350000
	3					5.0	1652.0	2896.67	19.60	10.271	0.00330000
	4					10.0	1703.8	3060.00	16.73	10.539	0.00240000
	5					5.5	1616.6	2890.00	18.38	9.072	0.00330000
	6					5.5	1720.1	3023.33	19.54	10.013	0.00310000
	7					4.0	1635.2	2930.00	17.85	9.515	0.00310000
	8					5.0	1634.6	2896.67	18.82	9.079	0.00330000
	1	1.0	6.0	30	0	4.5	1761.1	3070.00	16.67	12.034	0.00210000
	2					7.5	1694.6	3003.33	20.24	8.509	0.00330000
	3					7.0	1706.0	3013.33	17.89	9.197	0.00270000
	4					5.0	1749.6	3086.67	14.86	9.972	0.00210000
	5					5.0	1653.4	2926.67	18.16	9.338	0.00300000
	6					6.5	1705.8	3046.67	17.95	10.852	0.00290000
	7					5.0	1679.5	2986.67	14.92	11.560	0.00230000
	8					5.0	1666.8	2983.33	14.89	11.570	0.00110000
